# Occupational radiation exposure of radiologic technologists in Interventional neuroradiology

**DOI:** 10.1007/s00234-025-03807-7

**Published:** 2025-10-17

**Authors:** Anna Beckert, Christopher Kloth, Angela Kretschmer, Bernd Schmitz, Johannes Rosskopf

**Affiliations:** 1 Section of Neuroradiology, Bezirkskrankenhaus Günzburg, Günzburg, Germany; 2 Department of Diagnostic and Interventional Radiology, University Hospital Ulm, Ulm, Germany; 3 German Heart Center at TUM University Hospital, Technical University of Munich, Munich, Germany

**Keywords:** Occupational radiation exposure, Radiologic technologists, Interventional neuroradiology, Radiation protection

## Abstract

**Purpose:**

With the rising number of angiographic interventional procedures, occupational radiation exposure is becoming increasingly relevant. As even low doses may contribute to stochastic health effects, enhanced radiological protection measures are warranted. This study aimed to systematically quantify the radiation exposure of radiologic technologists under routine clinical conditions and in an experimental setting.

**Methods:**

Radiation dose exposure was monitored over one month using three official dosimeters placed at defined locations within the angiography suite, using the floor-mounted C-arm as a reference point. Dose values were extrapolated to estimate annual exposure. Additionally, experimental dose rate measurements were performed at eight locations and three height levels (foot, torso, eye) under standardized conditions using both standard and low-dose DSA protocols.

**Results:**

Official dosimeter readings averaged less than 2% of the reference value at the C-arm. The extrapolated annual occupational radiation exposure was low (0.44 mSv), with the highest value of 1.03 mSv near the door to the supply room. Experimental measurements revealed average radiation dose rates of 885 µSv/h; with a wide range from: 12 to 6109 µSv/h. Dose rates were more strongly influenced by the shielding effect of stationary protective equipment (reduction factor of 31) than by spatial distance. The highest radiation exposure occurred at foot level. Low-dose protocols reduced ambient radiation by an average of 23%.

**Conclusion:**

Occupational exposure remained well below legal thresholds but varied spatially. Stationary shielding and low-dose protocols proved most effective for dose reduction.

## Purpose

Optimization of occupational radiological protection in interventional procedures is a continuous process involving a wide range of factors [1, 2]. According to the 2023 publication of the International Commission on Radiological Protection (ICRP) [2] this process should include, first, effective collaboration between radiologists/radiologic technologists and medical physicists. Second, it requires the use of appropriate methodologies and technologies and, third, the implementation of well-structured organizational processes such as equipment performance testing, patient and occupational dose auditing, and protocol review.

It is assumed that optimization will only occur if all staff are properly trained in their roles [2]. In this context, the forthcoming, yet unpublished, ICRP publication titled “Practical Aspects in Optimization of Radiological Protection in Digital Radiography, Fluoroscopy, and CT” suggests the need for a practice-oriented guidance document that provides concrete instructions to support such training and implementation efforts.

Training specifically targeting radiation protection practices of radiologic technologists remains surprisingly rare, despite the fact that they are among the staff most frequently exposed to occupational radiation [3–6]. Admittedly, occupational annual doses between 1980 and 2020 among the U.S. Radiologic Technologists cohort comprising 43,823 participants were relatively low averaging 0.65 mSv [4]. Nevertheless, minimally invasive procedures performed under fluoroscopic guidance have experienced exponential growth, particularly in the field of neurointervention, with mechanical thrombectomy emerging as a key treatment strategy for acute ischemic stroke [7]. As a result, occupational radiation exposure is at risk of increasing if further optimization of radiological protection such as behavioral training is not implemented.

Exposure to low radiation dose i.e., below 100 mSv may also contribute to radiation-induced cancer [8–10]. According to the “linear no-threshold model” (LNT) even a single X-ray photon may be capable of initiating a carcinogenic process [11]. A recent meta-analysis published in 2024 confirmed that prolonged exposure to low-dose radiation over many years can lead to stochastic health effects [12]. Furthermore, the risk of radiation-induced cancer and leukemia is known to be influenced by several factors, including sex, age at exposure, and time since exposure [9, 13]. Moreover, for radiologic technologists, radiation exposure of the eye lenses increases the risk of lens damage or cataract formation [14, 15]. Given that the majority of radiological technologists are younger females enhanced radiological protection measures for this occupational group are urgently warranted.

Therefore, this prospective single center observational study aimed to systematically quantify the radiation exposure of radiologic technologists using both, official dosimetry under routine clinical conditions and experimental measurements of dose rate distribution at various locations within the angio-suite of a biplanar angiography system. The findings of this study were expected to serve as a foundation for evidence-based guidance to improve radiological protection practices and professional training.

## Methods

### Angiography suite setup

**Fig. 1:**
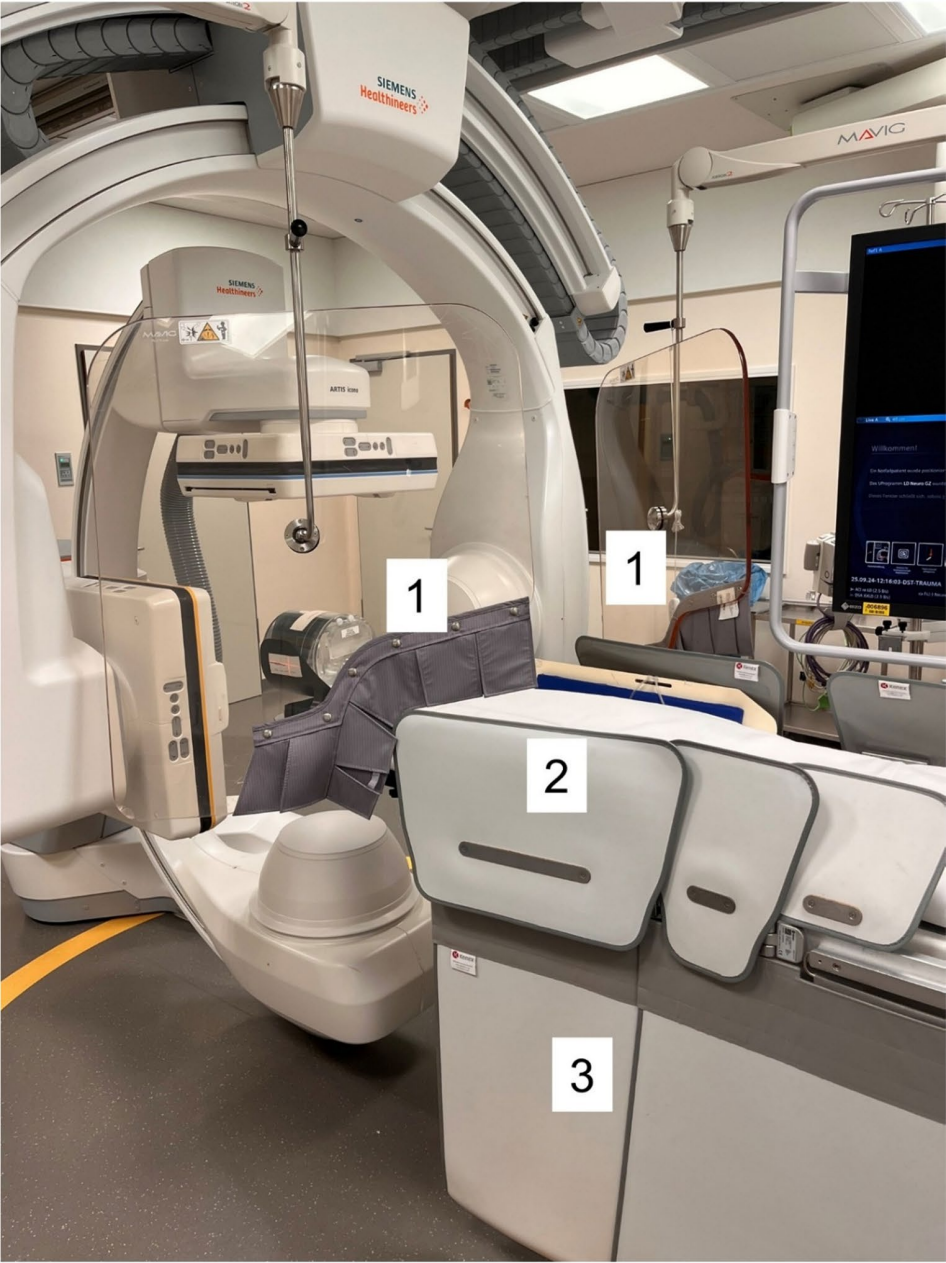
Angiography suite during the experimental setup. Biplanar Artis Icono System (Siemens Healthineers) and ceiling-mounted lead glass shields (1) positioned on the interventionalist’s and on anesthesiologist’s side. Lead lamellae above (2) and below (3) the table

At our institution, a biplanar Artis Icono system (Siemens Healthineers) is installed, featuring floor- and ceiling-mounted C-arms. The flat-panel detectors are positioned above the patient table and on the interventionalist’s side. Radiation protection is provided by lead lamellae above and below the table on both the interventionalist’s and the anesthesiologist’s side, as well as by a ceiling-mounted lead glass shield with a lead equivalent of 0.5 mm Pb, also positioned on the interventionalist’s and the anesthesiologist’s side (Fig. 1).

#### Setting a: routine clinical practice

Between January 8 and February 6, 2025, three different dosimeters were used to monitor radiation dose distribution under routine clinical conditions: (A) a whole-body dosimeter Hp(10), (B) an area dosimeter H*(10), and (C) an eye lens dosimeter Hp(3) (Table 1). All dosimeters are based on optically stimulated luminescence (OSL) technology. Hp(10) refers to the personal dose equivalent at 10 mm tissue depth (whole-body), Hp(3) to the personal dose equivalent at 3 mm (eye lens), and H*(10) to the ambient dose equivalent at 10 mm depth in a phantom representing the human body. Official dose reporting was conducted by MIRION Technologies – Dosimetry Services (Mirion Technologies (MGPI H&B) GmbH, Munich, Germany). The dosimeters used were the legally mandated dosimeters issued by the institutional radiation protection service.

**Table 1:** Official dosimeters

	Dosimeter	Measurement method	Dose quantity	Photon energy	Measurement range
A	whole-body dosimeter	OSL^1^	Personal dose equivalent Hp(10)	16 keV – 7 MeV	0.1–10 Sv
B	area dosimeter	OSL	Ambient dose equivalent H*(10)	16 keV − 1.25 MeV	0.05–1 Sv
C	eye lens dosimeter	OSL	Eye lens dose equivalent Hp(3)	16 keV – 7 MeV	0.1–1 Sv

^1^OSL = optically stimulated luminescence

**Fig. 2:**
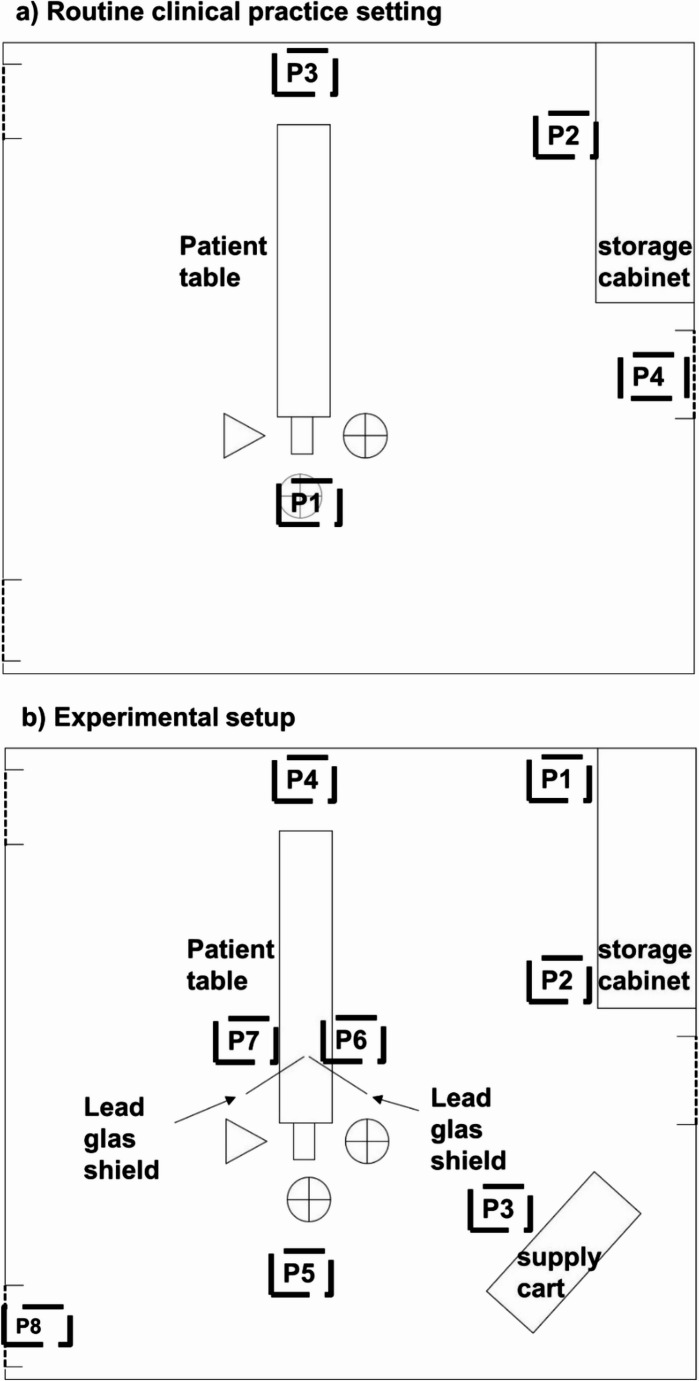
**a** Measurement positions of the official dosimeters in the angio-suite. P1: reference position at the C-arm. P2: routine working location of the radiologic technologists near a storage cabinet. P3: at the wall-mounted infusion flush system. P4: at the door leading to the adjacent supply room. **b** Experimental setup with directly readable dose rate monitor at various locations within the angio-suite (P1-P8). Measurements on both the interventionalist’s and the anesthesiologist’s side (P6, P7) performed for standard DSA protocol with and without the use of lead glass shields

The dosimeters were placed at defined locations within the angiography suite (Fig. 2a). For reference, dosimeters A, B, and C were mounted at a height of 108 cm ± 1 cm on the floor-mounted C-arm of the biplanar system (position 1, P1), at mid-height between the x-ray tube and the detector. In addition, further dosimeters A, B, and C were positioned in a distance of 420 cm from the reference point at the routine working location of the radiologic technologists near a storage cabinet (P2; height positioning: A: 88 cm; B: 107,5 cm; C: 160 cm). Dosimeters A and B, as well as B and C, recorded radiation doses at distances of 374 cm and 387 cm, respectively, from the reference point at the wall-mounted infusion flush system (P3) and a door leading to the adjacent supply room (P4). In summary, dosimeters A, B, and C at positions P2, P3 and P4 were used for environmental monitoring. A further eye lens dosimeter (C) was worn by the radiologic technologist during the intervention, attached to a surgical cap at the level of the left temple. No dose was measured at the protective apron.

#### Setting b: experimental setup

The experimental setup was designed to align with routine clinical conditions:

To simulate the presence of a human head, a water-filled cylindrical VCT QA Phantom (GE Healthcare) was positioned in the head shell and remained in the same place throughout all procedures. The phantom measured 20 cm in length with a diameter of 21.5 cm. The field size was set to 32 × 40 cm, corresponding to a standard posteroanterior and lateral projection. The source-to-detector distance was 85 cm. Biplanar acquisitions were performed with one arm oriented vertically and the other positioned at 90° (compare Fig. 1).

The distribution of the measurement positions in the angio-suite is shown in Fig. 2b. Measurements on both the interventionalist’s and the anesthesiologist’s side (P6, P7) were performed with and without the use of lead glass shields for standard DSA protocol. Moreover, the positions P1, P2, P6, P7, and partially P4 are protected by lead glass shields. The most distant measurement point was P8, located at the door to the control room, 450 cm away from the X-ray tube. Each position was measured at three different height levels: at floor level (feet), 126 cm (torso), and 160 cm (eye lens).

The LB 134 directly readable dose rate monitor (Berthold Technologies GmbH & Co. KG, Bad Wildbad, Germany) was used for experimental measurements. The monitor covers 0.1 µSv/h to 20 mSv/h, 50–1300 keV, and is calibrated to H*(10) with 0.625 µSv/h/cps and 0.07 cps background. According to typical specifications of comparable portable dose rate monitors, the measurement uncertainty can be assumed to be approximately ± 15–20% under reference conditions. The measured dose rate given in µSv/h is recorded using the counter-timer mode.

The acquisition protocol comprised a 20-s biplanar DSA series with a fluoroscopy time of 20.16 s and a mean tube voltage of 70.86 kV (SD 1.69). Image acquisition rate was 2.5 images per second. The cumulative DAP of both planes averaged 1,569 µGy·m² (SD 198). In the low-dose DSA protocol, the tube voltage was 69.2 kV (SD 1.9), resulting in a cumulative DAP of 1,052 µGy·m² (SD 149).

Fluoroscopy was not included, as its contribution to the overall dose is typically negligible and was therefore not considered.

### Statistical analysis

For the routine clinical practice (**setting a**) the measured radiation dose at each position was compared to the reference measurement at the C-arm using both absolute and relative metrics. Extrapolation of annual doses was based on the observation that, in the preceding year, a radiological physician attended a median of 73 angiographic procedures (Q1: 69; Q3: 81). Further, radiation exposure was extrapolated to one year for a single radiologic technologist, assuming that occupational radiation exposure correlates with the Dose area product (DAP). The extrapolation was based on a median DAP of 6,769 cGy·cm² and 57 procedures during the observation month, as well as 73 angiographic procedures assisted by a single radiologic technologist in 2024. Accordingly, a radiographer performing 73 angiographies would be associated with a cumulative DAP of 494,137 cGy·cm² per year. The readings from official dosimeters were scaled to this annual DAP. The resulting extrapolated doses in mSv were compared to the ICRP-recommended annual limits for occupational radiation exposure [16].

Radiation dose rates in the experimental setup (**setting b**) were analyzed using both absolute and relative metrics at both individual and grouped levels, including comparisons between shielded and unshielded positions, as well as variations in spatial distance, measurement height, and DSA protocol.

## Results

### Setting a: routine clinical practice

#### Measured radiation dose compared to reference at the C-arm

The average measured radiation dose was 1.6% of the reference value at the C-arm (P1) (dosimeter A: 1.5%, B: 1.6%, C: 1.5%). At the door leading to the adjacent supply room (B, P4) the highest dose level of 1.03 mSv was measured. Thus, the radiation dose at position P4 (dosimeter B) was on average 2.4 times compared to the other positions P2 and P3 (1.03 mSv vs. 0.44 mSv). The average radiation dose levels were fairly evenly distributed between positions P2, and P3 with differences of less than a factor of 2. The detailed measurement data are shown in Table 2.

**Table 2:** Measurement data during routine conditions

Measurement position	Measured radiation dose	Extrapolated annual occupational radiation exposure
P1 ^1^	A: 22.80, B: 39.05, C: 28.00^6^	A: 28.37, B: 48.59, C: 34.85
P2 ^2^	A: 0.40, B: 0.62, C: 0.30	A: 0.50, B: 0.77, C: 0.37
P3 ^3^	A: 0.30, B: 0.25	A: 0.37, B: 0.31
P4 ^4^	A: 0.30,**B: 1.03**, C: 0.60	A: 0.30, B: 1.28, C: 0.75
left temple ^5^	A: 0.50, B: 0.77, C: 0.40	A: 0.50, B: 0.77, C: 0.50

^1^reference position at the C-arm

^2^routine working location of the radiologic technologists near a storage cabinet

^3^at the wall-mounted infusion flush system

^4^at the door leading to the adjacent supply room

^5^eye lens dosimeter worn by the radiologic technologist during the intervention attached to a surgical cap at the level of the left temple

^6^A = whole-body dosimeter. B = area dosimeter C = eye lens dosimeter. Due to the limited number of dosimeters available, it was not feasible to perform measurements at all positions within the room. This also explains the gaps in the corresponding table. The highest value 1.03 mSv (P4, B) is highlighted in black

unit: mSv

### Extrapolated annual occupational radiation exposure

The extrapolated annual occupational radiation exposure measured by the whole-body dosimeter (A) averaged 0.44 mSv for positions P2 and P3, indicating a much lower level compared to the ICRP-recommended annual limit of 20 mSv for occupational exposure. Individual extrapolated annual exposure values (in mSv) are presented in the final column of Table 2.

#### Setting b: experimental setup

Experimental measurements revealed average radiation dose rates of 885 µSv/h, ranging from 12 µSv/h at the wall-mounted infusion flush system (P4, foot level) to 6109 µSv/h at the unshielded anesthesiologist’s side (P7*, torso level) (Table 3).

**Table 3:** Measurement data from the experimental setup

	standard protocol			low-dose protocol		
	feet	torso	eye	feet	torso	eye
P1	542	127	129	339	79	78
P2	696	270	200	413	161	122
P3	1452	1355	1236	782	837	764
P4	23	877	944	12	522	579
P5	60	104	260	32	62	161
P6	2371	280	284	2143	238	230
P6*		4962	4206			
P7	3308	196	167	3429	240	202
P7*		6109	5125			
P8	115	290	314	58	230	260

*at positions P6 and P7, both shielded and unshielded (*) data were acquired. unit: µSv/h

When utilizing all available stationary protective equipment, the average dose rate was 23% higher in the standard protocol compared to the low-dose protocol (650 µSv/h ± 807 µSv/h (95% CI: 309–991µSv/h) vs. 499 µSv/h ± 768 µSv/h (95% CI: 174–823 µSv/h).

The highest exposure was consistently observed at foot level, compared to torso and eye level, for both protocols (+ 59% in the standard protocol and + 67% in the low-dose protocol).

**Fig. 3:**
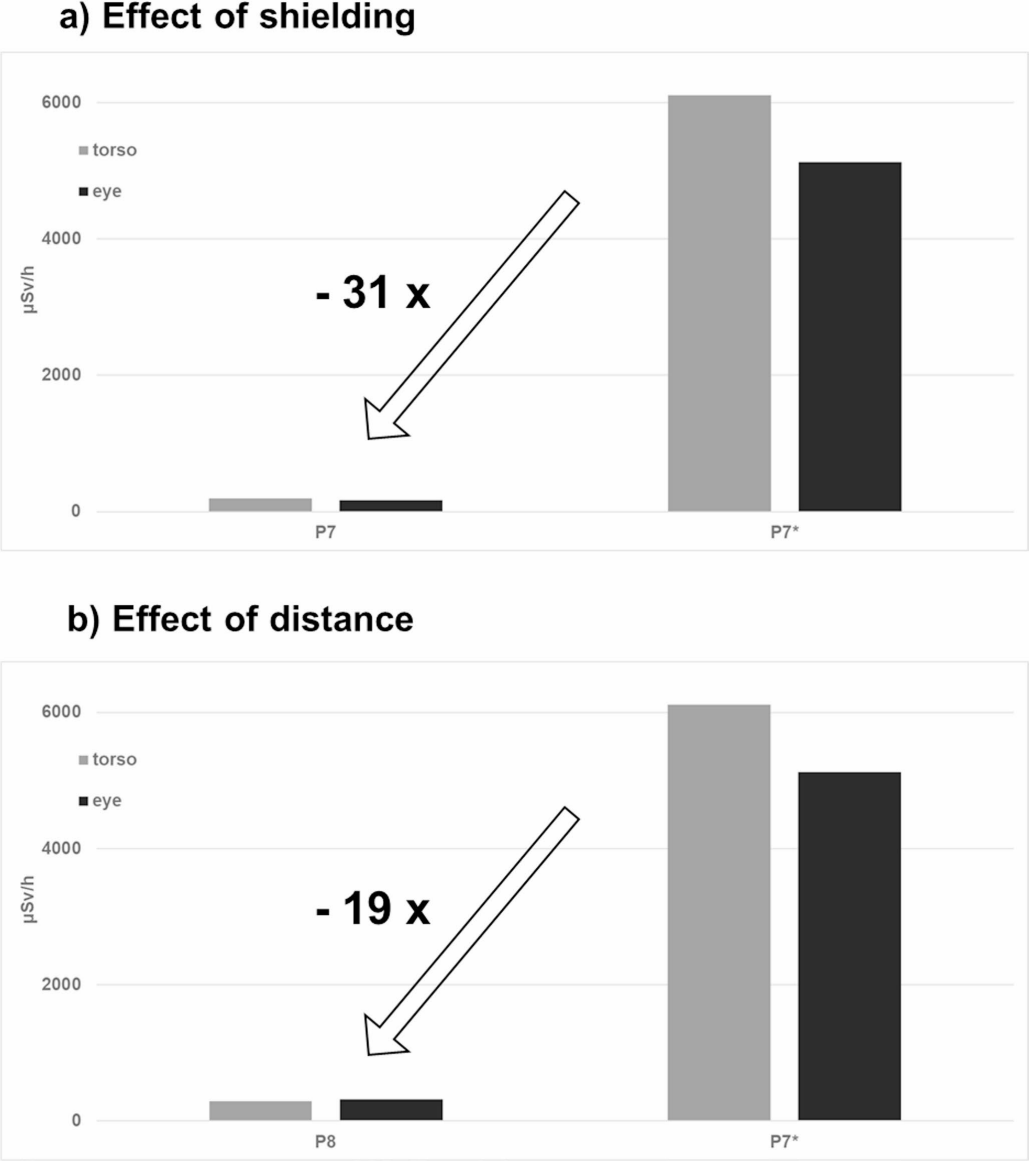
Radiation exposure reduction: (**a**) with lead glass shielding (up to factor 31; shielded P7 vs. unshielded P7*) and (**b**) with distance alone (up to factor 19; P8 at the control room door vs. P7* unshielded on the anesthesiologist side)

The use of lead glass shields reduced dose rates for torso and eye by a factor of up to 31 at position P7 and P7*, respectively. By comparison, increasing distance alone (P8 vs. P7*) yielded a maximum factor of 19. Figure 3 illustrated the comparison of shielding and distance using bar plots.

## Discussion

This study quantified radiation exposure of radiologic technologists during angiographic procedures. Despite a very low extrapolated annual dose of 0.44 mSv, spatial variations were evident, with the highest value (1.03 mSv) measured near the supply room door (P4, area dosimeter B). Experimental data showed dose rates ranging from 12 to 6109 µSv/h, with the highest exposure at foot level. Stationary shielding reduced dose rates to the torso and the eye by a factor of 31. Low-dose DSA protocols reduced ambient radiation by an average of 23%.

Measurement and simulation for estimating potential exposure to staff during fluoroscopic procedures are widely used in various medical fields, such as interventional radiology, interventional cardiology, and interventional endoscopic retrograde cholangiopancreatography (ERCP) [17]. These data from different scenarios can help develop strategies to minimize radiation exposure risks in clinical settings and serve as an educational resource for local staff.

However, particularly in neuroradiology, greater fluctuations in radiation exposure are observed due to the wide variety of interventions, which differ in complexity and the materials use; unlike the more standardized procedures in cardiology or internal medicine [6, 18].

Rigatelli et al. found that radiologists shorter than 165 cm recorded higher dosimeter readings compared to their taller colleagues, despite wearing the dosimeter in the same position [19]. This may be attributed to the distribution of scatter radiation, which tends to affect the extremities more significantly - a finding that aligns with our own results concerning radiation exposure to the feet [20]. Additionally, conventional lead aprons typically do not extend to cover the feet. Supporting this, Otomo et al. demonstrated that lower measurement positions were associated with higher spatial radiation doses during mobile radiography [21]. Similarly, Kim et al. reported a vertical gradient of exposure from the eyes down to the feet in radiographic examinations [22]. Our extrapolated annual dose of 0.44 mSv is consistent with the findings of Milder et al., who reported a median annual dose of 0.65 mSv (interquartile range, 0.60–1.40 mSv; 95th percentile, 6.80) [4].

For optimal protection, the greatest possible distance from the radiation source should be maintained, and the maximum available protective shielding should be utilized [23]. Distance is a particularly effective means of reducing exposure, as radiation dose decreases with the square of the distance from the source [23]. The unexpectedly high dose recorded at P4 (1.03 mSv/month in H*(10)) might therefore unlikely to reflect direct exposure, given its peripheral location at the door. The finding might be attributable to scattered radiation accumulating in this geometric position or, less likely, to leakage from the X-ray system. In addition, our results emphasize that significant dose reduction can also be achieved through the consistent use of low-dose examination protocols.

To enhance individual protection against radiation exposure, the simulation of dose levels during interventions is an emerging tool that supports dose reduction strategies and promotes practical dose management as part of hands-on training. It enables institution-specific improvements and helps optimize the behavior of individual trainees and radiologic technologists [24]. A relevant concern is that the weight of lead aprons often leads to discomfort, fatigue, and musculoskeletal issues, particularly back pain [24–26]. Over time, and with an increasing number of daily interventions, reducing background radiation exposure may allow for lighter aprons with less shielding, potentially improving wearer comfort without compromising safety. In this context, manufacturers are also called upon to improve shielding of scatter radiation directly at the patient table in order to better protect medical personnel.

Overall, and in line with current guidelines, we recommend that radiologic technologists receive continuous training throughout their careers, along with regular instruction in radiation protection and dose management, to minimize lifetime radiation exposure [24]. Both the Society of Interventional Radiology (SIR) and the Cardiovascular and Interventional Radiology Society of Europe (CIRSE) emphasize the importance of radiation protection in interventional radiology and highlight the need for appropriate staff education and training in their guidelines [27–29]. This aligns with the recommendations of the International Atomic Energy Agency (IAEA) and the International Commission on Radiological Protection (ICRP), which state that institutions and employers are responsible for providing workers with the necessary information, guidance, and training to ensure radiation safety [30].

To put it in a nutshell, the findings of this study highlighted that comparatively higher radiation exposure can occur even at greater distances from the x-ray tube, such as at the entrance doors of the angiosuite (P8). As a practical consequence, efforts should be made to keep these doors consistently closed during radiation exposure. Furthermore, the study emphasizes once more the protective effect of shielding. Radiologic technologists should therefore consistently remain within the shadow of shielding inside the angiosuite, even when positioned farther away from the radiation source.

This study was not without limitations. First, the data collection in a single-center was limited to a period of one month, rather than a full working year. As a result, the annual radiation exposure values were extrapolated based on this short-term data, which may not fully capture long-term variability. Moreover, due to the pulsed nature of the radiation, instantaneous dose rates during the pulses may have exceeded the rated measurement range of the LB 134 monitor. Therefore, measured values at high dose rates should be interpreted with caution, as they may be subject to inaccuracy. Furthermore, in the experimental setup only the patient’s head was simulated, meaning attenuation by the rest of the body was not accounted for. Also, no repeated measurements at identical positions were performed; therefore, quantitative estimates of measurement error are not available. Nevertheless, as the dosimeters and the LB 134 dose rate monitor are commercially available and well-established devices, only low to moderate variability can be assumed. Individual exposure can vary significantly depending on each technologist’s working hours, the number and type of procedures performed, and their specific roles during interventions. No behavioral analysis was applied. Furthermore, patient radiation exposure during the procedures was not separately evaluated. Including patient dose data in future studies could offer valuable insights into correlations between patient and staff exposure. Finally, we did not assess individual differences in radiation protection practices or adherence to dose monitoring protocols among radiologic technologists. Variability in personal behaviour and compliance with safety measures may have influenced exposure outcomes.

## Conclusion

In this study, radiation exposure for radiologic technologists remained well below legal limits but varied depending on room position. The most effective dose reduction was achieved through the use of stationary shielding and low-dose DSA protocols, while distance alone had a smaller impact. These findings highlight the importance of structured radiation protection strategies and underscore the need for ongoing training and protocol optimization to ensure consistent safety in clinical practice.

## Data Availability

No datasets were generated or analysed during the current study.

## Abbreviations

ICRP: International Commission on Radiological Protection
OSL: Optically Stimulated Luminescence
DAP: Dose Area Product
DSA: Digital Subtraction Angiography
